# A comprehensive review of Hungarian futures studies in light of international journal articles

**DOI:** 10.1186/s40309-022-00201-x

**Published:** 2022-06-23

**Authors:** Erzsébet Nováky, Tamás Kristóf

**Affiliations:** 1 Institute of International, Political and Regional Studies, Corvinus University of Budapest, Fővám tér 8, Budapest, H-1093 Hungary; 2 Institute for the Development of Enterprises, Corvinus University of Budapest, Fővám tér 8, Budapest, H-1093 Hungary

**Keywords:** Futures studies, Systematic literature review, Scientometrics, Development history, Hungary

## Abstract

This article offers an overview of the evolution of Hungarian futures studies via a systematic literature review of articles with at least one Hungarian futurist (co-)author, published in high-ranking international or English-language Hungarian journals. The review reveals how researchers from a relatively small European country, where central planning had been applied for decades, have made their way to the pages of prestigious international journals and disseminated their results in a diverse range of articles to the global research community. The number of these publications has increased decade by decade. Results of statistical-based literature review demonstrate that research period and research topic are in strong association with the quality of journal articles, yet scientometric features of Hungarian futurist (co-)authors are not significant in this aspect. However, spectacular clustering of articles can be accomplished based on the citation statistics of Hungarian futurist (co-)authors.

## Introduction

Futures studies (FS) began in Hungary in 1968, the same year when the Club of Rome was founded. Its evolution was not smooth; its scientific nature was often questioned. However, the quality of central planning required a significant improvement, and thus, FS was perceived as a new approach providing appropriate methods. While Hungarian FS initially had to define and defend its position and idiosyncrasies in that context, the leading scholars set a broader objective for themselves, and hence for this new school of thoughts: to contribute to the development of the theoretical and methodological knowledge base that can be mobilized when exploring the future. In other words, their aim was to enrich the international futures literature, join the international futures network, and conduct research and education activities in a mutually reinforcing manner. Time has shown that these objectives have been the right ones. In 1976, FS became a recognized discipline at the Hungarian Academy of Sciences (HAS), and it has been involved in doctoral programs since 2009.

Hence, Hungarian FS has gone through a development path different from the classic three-phase periodization of Western FS, and initially followed the features of the Soviet model [138]. This deterioration is derived from the deterministic conception of traditional Marxism. The priorities of FS were subjected to long-range forecasting and strategic planning [10]. FS was part of a centralized national development program focused on economic growth and a policy-making guide.

The terms “futures studies” and “futures research” are used in a broad sense in this article, that is, as an alternative expression to forward-looking activities (FLA) or prospective analyses that can be conducted in various forms for pursuing different purposes. The best-known forms include forecasting, critical (or key) technologies, foresight, long-range planning, and indicative national planning [43].

Our review of articles having at least one Hungarian co-author, published in high-ranking international journals, reveals the diversity of Hungarian FS. Hungarian researchers have dealt with a broad spectrum of futures issues, incorporating sustainability, socio-economic development, globalization, regional development, health, education, strategic foresight by businesses, and future orientation examinations, together with underpinning science, technology, and innovation policies. Clarification of theoretical and methodological problems has always been a prime concern. Each article was elaborated in the spirit of futures concept and futures methodology, demonstrating that FS has contributed to effectively handle the future challenges of diverse research fields.

Beyond publishing in various scientific journals, Hungarian futurists have been actively taking part in the Executive Board of World Futures Studies Federation (WFSF), completed several international and domestic foresight projects, operated the local association of the Club of Rome, and have a local node in The Millennium Project, and Hungarian young futurists have won in all the four categories of the Association of Professional Futurists (APF) awards. Hungary organized two world conferences (in 1991 and 2005), one regional discussion (in 1987), and four summer courses (in 1999, 2001, 2003, and 2005) for the WFSF, as well as the Technology Foresight Summit in 2007 for the United Nations Industrial Development Organization (UNIDO). Hungarian futurists actively participated in the work of COST A22 action (Foresight methodology – Exploring new ways to explore the future, 2003–2007) that also contributed to the appreciation of achievements of Hungarian futurists.

This paper can be positioned in academic literature as a review article and at the same time a national case study. Its aim is to provide instructive examples of practices together with lessons learned that can be applied in other countries. Similar articles with similar objectives have been already published inter alia for Brazil [8], Korea [45], Germany [46], Ireland [91], Sweden [123], and Finland [143]. Evolution of FS has been in-depth discussed in several articles, see i.e. *Slaughter* [137], *Masini* [93], *Kuosa* [90], *Son* [138], or *Schultz* [130]. Hideg [53] provided a historical evaluation of international mainstream futures field through its evolution from forecasting across evolutionary and critical futures studies and foresight to integral futures.

The objective of this article is to provide a systematic literature review of international journal articles (co-)authored by Hungarian futurists. Features of the reviewed articles are evaluated using bivariate and multivariate statistical methods within the framework of a hypothesis examination. The intention is to reveal the descriptive variables of (co-)authors and articles indicating a significant statistical relationship with the quality of articles expressed by journal ranking. Results demonstrate that research period and research topic are in strong association with the quality of journal articles; however, scientometric features of Hungarian futurist (co-)authors are not significant in this aspect.

The article is structured as follows. The first section describes the research methodology to complete the literature review including the features of data collection and variable specification. The second section evaluates the results of the review using distribution analysis, bivariate statistical association analysis, multivariate decision trees, and visual clustering techniques. The subsequent section tells a story of the evolution of Hungarian FS based on the reviewed articles. The concluding section summarizes the results of the findings.

## Research methodology

**Fig. 1:**
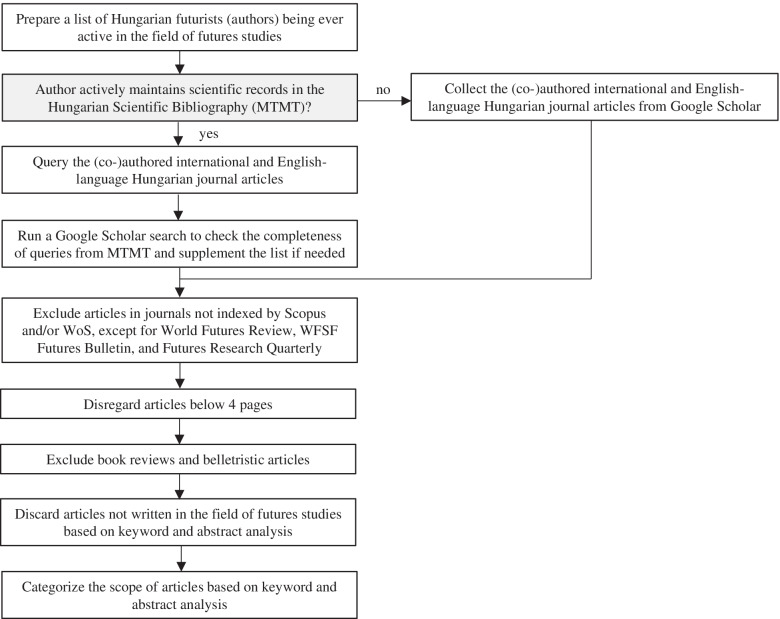
The process of conducting the literature review

This review covers articles (co-)authored by at least one Hungarian futurist (or other scholars engaged in FLA), published in international or Hungarian journals in English in this domain, indexed by Scopus and/or Web of Science (WOS). In addition, articles published in World Futures Review, World Futures Studies Federation – Futures Bulletin, and Futures Research Quarterly are also included, due to the significance of these journals. Figure 1 presents the steps of the data collection process.

The primary source of data collection was the Hungarian Scientific Bibliography (MTMT, Magyar Tudományos Művek Tára in Hungarian), where all active Hungarian researchers are obliged to list their publications and citations. Initially, a list was assembled to collect authors who have been ever active in the field of FS, broadly defined, concentrating on academic and higher education affiliations and known FS practitioners. Then, the (co-)authored journal articles were queried from MTMT. To crosscheck the completeness of these publication records, we run a Google Scholar search for known Hungarian authors in the field of FS. This step was of utmost importance for Hungarian futurists who passed away and whose MTMT records have not been actualized for a long time.

In line with the accustomed journal article quality standards, the minimum length of the articles was set as four pages. Book reviews and belletristic articles were excluded from selection. In order to ensure that the considered articles were written in the field of FS, a keyword and abstract analysis was performed. Preferred words, concepts, and methodologies underpinned the process, such as future, foresight, forecasting, backcasting, scenarios, or development trends. It also served for putting the articles into context to elaborate the story of the evolution of Hungarian FS presented in the “The story of FS in Hungary in light of international journal articles” section.

Since Hungary is a relatively small country, and the research community working in this field is also a small one, compared to other domains, it is unlikely that an important article authored by a Hungarian futurist has remained uncharted by the authors. Hence, we assume that our review is essentially a complete one. Due to its nature, a comprehensive review cannot present the results published in these articles in-depth. Readers interested in detailed observations, arguments, and conclusions presented in these articles are advised to read the respective publications.

In order to facilitate the statistical analysis of the collected articles, the following descriptive variables have been defined for each article:

1. Number of authors
2. Year of publication
3. Ranking of the journal
4. Research topic
5. Feature of FLA
6. Total number of publications of the best^1^ Hungarian futurist (co-)author
7. Total number of journal article publications of the best Hungarian futurist (co-)author
8. Total number of independent citations of the best Hungarian futurist (co-)author
9. Hirsch index of the best Hungarian futurist (co-)author
10. Did the best Hungarian futurist (co-)author ever (co-)author a FS article in a Q1 journal?
11. Did the best Hungarian futurist (co-)author ever (co-)author a FS article in Futures?

For further analysis, the continuous variables (1, 2, 6, 7, 8, and 9) have been categorized, presented in the “Results of review” section. To identify relationships, first in a bivariate approach, associations between the variables have been measured using the Cramér V statistics. It is based on Pearson’s chi-squared statistic, giving a value between 0 and 1 [11].

Then, a machine learning-based multivariate Classification and Regression Tree (CART) model has been developed to explain the simultaneous effects of the relevant variables on the publication quality of Hungarian futurists. The target variable has been specified by the ranking of journal articles. Since the target variable has more than two outcomes, the multinomial CART algorithm has been applied [7].

Finally, a machine learning-based visual clustering has been performed on the database using Self-Organizing Maps (SOM) to explore the relevant grouping variables and clusters of the publications. SOM belongs to the feedforward type neural network family having unsupervised learning features. During the self-organizing process, original data has been transformed into a topological representation by reducing the multidimensional data into a two-dimensional map [74]. It has been attempted to detect clusters among the examined articles with the aim of finding relevant clusters according to the citation statistics of authors.

Empirical research of the literature review is supported by the examination of the following hypotheses:

- *Hypothesis 1*: Ranking of journal articles where Hungarian futurists can publish is determined by research topic, feature of FLA, and the period of publishing.
- *Hypothesis 2*: Ranking of journal articles where Hungarian futurists can publish is determined by the scientometric parameters of the “best” Hungarian (co-)author of a given article.
- *Hypothesis 3*: Visual clustering of journal articles where Hungarian futurists were able to publish can be accomplished by considering the independent citation profile of the “best” Hungarian (co-)author of a given article.

## Results of review

Altogether, 136 articles met the predefined requirements, listed above in the “Research methodology” section. Table 1 presents the number of articles by the period of their publishing. In the 1970s and 1980s, publications mostly appeared in domestic journals and books. At that period, futures articles mainly focused on curiosity, rather than on presenting scientific results. Methodological development, however, was already a prime objective for the Hungarian FS community. Before the 1990s, those who wanted to publish in western international journals had to receive permission from the Ministry of Home Affairs. Hence, those who worked and researched abroad some years had a better chance to publish. It meant a substantial restriction in that era for Hungarian futurists to disseminate results in internationally recognized journals. The first noticeable publication boom in high-ranking international journals occurred in 1994, after the 25th jubilee of the discipline in Hungary.

**Table 1:** Distribution of articles by the period of publishing

Period	Number of publications	Average number of authors
Before 1990	7	1.143
1990–1999	23	1.565
2000–2009	26	1.462
2010–2019	60	2.283
Since 2020^*^	20	3.550
**Total**	**136**	**2.132**

^*^Data collection was closed on 31 December 2021

As a result of expanding research activities, the number of articles increased significantly in the 2010s, which was the most fruitful decade for Hungarian futurists so far. The first 2 years of the 2020s appear to be at least as promising. The three most cited articles were *Havas et al.* [42] with 168, *Meskó et al.* [95] with 126, and *Havas* [37] with 101 independent citations received until 31 December 2021. It can also be observed that the average number of authors has been significantly increasing throughout the analyzed period.

By analyzing the abstracts and keywords of the articles, we classified the papers by their main themes, beyond the applied futures concepts and methods. Concerning the research topics of the articles, 17.6% of them can be regarded as methodological papers, 14.0% as papers on sustainability, and clarification of theoretical considerations was also an important research topic (11.0%). Table 2 presents the breakdown of the articles by their main research issue. The average number of authors is diverse per research topic. All the articles in the topic of globalization/international economy, together with the political and economic transition, were authored by a single Hungarian futurist, whereas articles in the field of sustainability were written by average 3.474 authors.

**Table 2:** Distribution of articles per research topic

Research topic	Number of publications	Average number of authors
Methodology	24	2.000
Sustainability	19	3.474
Theory	15	1.733
Future orientation examinations	13	2.077
Globalization/international economy	13	1.000
Future of health	10	2.100
Future of higher education	10	2.400
Science, technology, and innovation policy	9	2.000
Corporate future	9	2.889
Political and economic transition	6	1.000
Review article	5	1.800
Regional foresight	3	2.000
**Total**	**136**	**2.132**

In a different approach, the articles have been categorized according to their features of FLA, namely whether the articles reflect forecasting, foresight, or general FS characteristics. Table 3 demonstrates that more than half (51.5%) of the articles can be regarded as foresight-based publications, followed by general FS (29.4%) and forecasting-driven articles (19.1%). The average number of authors is substantially higher than average in the case of foresight articles.

**Table 3:** Distribution of articles by their features of FLA

Feature of FLA	Number of publications	Average number of authors
Forecasting	26	1.808
Foresight	70	2.629
General FS	40	1.475
**Total**	**136**	**2.132**

The 136 articles were published in 64 different journals. The concentration of the articles coincides with the prestige of journals in the field of FS (*Futures*, *Journal of Futures Studies*, *Technological Forecasting and Social Change*, *European Journal of Futures Research*, *Foresight*, *World Futures Review*). Besides international journals, social sciences and management journals published in Hungary in English, indexed in Scopus or WOS, have also been important publication outlets for Hungarian researchers (Society and Economy, Acta Oeconomica, Periodica Polytechnica – Social and Management Sciences). Table 4 shows the distribution of the articles per journal.

**Table 4:** Distribution of articles by the publication outlet

Journal	Number of publications	Average number of authors
*Futures*	20	2.200
*Society and Economy*	19	1.650
*Journal of Futures Studies*	8	2.500
*Technological Forecasting and Social Change*	6	1.833
*Acta Oeconomica*	6	1.333
*European Journal of Futures Research*	3	4.333
*Foresight*	3	1.667
*European Integration Studies*	3	1.333
*Periodica Polytechnica – Social and Management Sciences*	3	1.000
*Sustainability*	2	4.000
*World Futures Review*	2	2.500
*World Futures Studies Federation – Futures Bulletin*	2	1.000
*American Behavioral Scientist*	2	1.000
*Journal of Globalization Studies*	2	1.000
*Systems Research and Behavioral Science*	2	3.000
*Journal of Cleaner Production*	2	3.500
*Journal of Risk and Financial Management*	2	1.500
*Interdisciplinary Description of Complex Systems*	2	1.000
*Entrepreneurial Business and Economics Review*	2	2.000
Others (one article in a single journal)	45	2.467
**Total**	**136**	**2.132**

A more comprehensive picture can be gained about the quality of publication outlets by analyzing the distribution of actual journal ranking categories (see Table 5). 36.0% of the papers were published in Q1, and 23.5% respectively in Q2 and Q3 journals. In total, 111 of the 136 articles were (co-)authored by a Hungarian futurist who ever managed to publish a Q1 article in the topic of FS and 74 articles were (co-)authored by a Hungarian futurist who ever managed to publish at least one article in *Futures*.

**Table 5:** Distribution of articles by journal ranking categories

Journal ranking category	Number of publications	Average number of authors
Scopus Q1	49	2.490
Scopus Q2	32	2.500
Scopus Q3	32	1.656
Scopus Q4	5	1.000
WOS (not indexed by Scopus)	13	1.538
The three exceptional journals	5	2.000
**Total**	**136**	**2.132**

For further analysis, the Scopus Q4, the WOS (not indexed by Scopus), and the three exceptional journals were integrated into one category. A categorical variable was created from the number of authors indicating one, two, three, or at least four authors. In addition, the scientometric measures of authors were classified into the following categories. When creating the intervals, it was considered to prevent concentration and provide proper distribution.

- Total number of publications: ≤80; 81–200; 201–330; >330
- Total number of journal article publications: ≤20; 21–50; 51–99; >99
- Total number of independent citations: ≤150; 151–400; 401–800; >800
- Hirsch Index: ≤6; 7–10; 11–14; >14

Table 6 presents the results of pairwise statistical relationship examinations between the categorical variables. It is not surprising that the above four scientometric features (CAT6, CAT7, CAT8, and CAT9) have a strong (0.01 level) association with one another. Research topic (CAT4) has a significant association with each variable, which is also the case for the feature of FLA (CAT5) except for the last two variables, demonstrating that forecasting, foresight, or general FS themes have no relationship with the Scopus quality of publication outlets. The period of publication (CAT2) has a significant relationship with each variable, except for CAT11, indicating that publication activity in-time does not relate to the fact whether a Hungarian futurist ever (co-)authored an article in Futures.

**Table 6:** Association between the categorical variables using the Cramér V statistics and *p*-values

	CAT1	CAT2	CAT3	CAT4	CAT5	CAT6	CAT7	CAT8	CAT9	CAT10	CAT11
CAT1		0.250^*^ (0.012)	0.170 (0.226)	0.421^**^ (0.000)	0.270^**^ (0.003)	0.193 (0.085)	0.213^*^ (0.030)	0.171 (0.220)	0.234^**^ (0.008)	0.310^**^ (0.000)	0.396^**^ (0.000)
CAT2	0.250^*^ (0.012)		0.308^**^ (0.000)	0.371^**^ (0.002)	0.280^*^ (0.019)	0.332^**^ (0.000)	0.474^**^ (0.000)	0.334^**^ (0.000)	0.262^*^ (0.021)	0.269^*^ (0.043)	0.086 0.907)
CAT3	0.170 (0.226)	0.308^**^ (0.000)		0.379^**^ (0.004)	0.239^*^ (0.016)	0.161 (0.304)	0.199 (0.062)	0.189 (0.103)	0.116 (0.789)	0.412^**^ (0.000)	0.091 (0.773)
CAT4	0.421^**^ (0.000)	0.371^**^ (0.002)	0.379^**^ (0.004)		0.561^**^ (0.000)	0.551^**^ (0.000)	0.281^**^ (0.006)	0.488^**^ (0.000)	0.518^**^ (0.000)	0.384^*^ (0.044)	0.626^**^ (0.000)
CAT5	0.270^**^ (0.003)	0.280^*^ (0.019)	0.239^*^ (0.016)	0.561^**^ (0.000)		0.288^**^ (0.001)	0.290^**^ (0.001)	0.247^*^ (0.011)	0.277^**^ (0.002)	0.164 (0.161)	0.095 (0.544)
CAT6	0.193 (0.085)	0.332^**^ (0.000)	0.161 (0.304)	0.551^**^ (0.000)	0.288^**^ (0.001)		0.518^**^ (0.000)	0.518^**^ (0.000)	0.461^**^ (0.000)	0.165 (0.297)	0.542^**^ (0.000)
CAT7	0.213^*^ (0.030)	0.474^**^ (0.000)	0.199 (0.062)	0.281^**^ (0.006)	0.290^**^ (0.001)	0.518^**^ (0.000)		0.502^**^ (0.000)	0.587^**^ (0.000)	0.405^**^ (0.000)	0.376^**^ (0.000)
CAT8	0.171 (0.220)	0.334^**^ (0.000)	0.189 (0.103)	0.488^**^ (0.000)	0.247^*^ (0.011)	0.518^**^ (0.000)	0.502^**^ (0.000)		0.629^**^ (0.000)	0.399^**^ (0.000)	0.254^*^ (0.033)
CAT9	0.234^**^ (0.008)	0.262^*^ (0.021)	0.116 (0.789)	0.518^**^ (0.000)	0.277^**^ (0.002)	0.461^**^ (0.000)	0.587^**^ (0.000)	0.629^**^ (0.000)		0.371^**^ (0.000)	0.480^**^ (0.000)
CAT10	0.310^**^ (0.000)	0.269^*^ (0.043)	0.412^**^ (0.000)	0.384^*^ (0.044)	0.164 (0.161)	0.165 (0.297)	0.405^**^ (0.000)	0.399^**^ (0.000)	0.371^**^ (0.000)		0.518^**^ (0.000)
CAT11	0.396^**^ (0.000)	0.086 (0.907)	0.091 (0.773)	0.626^**^ (0.000)	0.095 (0.544)	0.542^**^ (0.000)	0.376^**^ (0.000)	0.254^*^ (0.033)	0.480^**^ (0.000)	0.518^**^ (0.000)	

*CAT1* number of authors, *CAT2* period of publication, *CAT3* journal ranking, *CAT4* research topic, *CAT5* feature of FLA, *CAT6* total number of publications, *CAT7* total number of journal article publications, *CAT8* total number of independent citations, *CAT9* Hirsch Index, *CAT10* ever co (authored) FS article in Q1 journal?, *CAT11* ever co (authored) FS article in *Futures*?

*Significant at 0.05 level

**Significant at 0.01 level

In order to test the validity of hypothesis 1, the statistical associations of CAT2, CAT4, and CAT5 are evaluated with CAT3. Based on the Cramér V values, it can be concluded that both the period of publication and the research topic are significant at 0.01 level, and the impact of the research topic is stronger. Results are logical from a professional viewpoint, as before 2000 most articles were published in Q3 journals, and ranking distribution has become more favorable afterwards. The quality of articles significantly varies by research topic. Most articles (co-)authored by Hungarian futurists were successfully published in Q1 journals in the fields of sustainability, methodology, future orientation examinations, theory, and future of health; however, so far, no Hungarian futurist has managed to publish in a Q1 journal in the topic of corporate future, regional foresight, and globalization/international economics. Hence, *hypothesis 1 is accepted*.

In order to test the validity of hypothesis 2, the statistical associations of CAT6, CAT7, CAT8, and CAT9 are evaluated with CAT3. Cramér V statistics suggest that neither scientometric variable shows significant association with journal ranking. Results demonstrate that publication and citation backgrounds do not significantly influence the quality of published journal articles. It means that Hungarian futurists with a lower track record can publish in high-ranking journals, and vice versa.

**Fig. 2:**
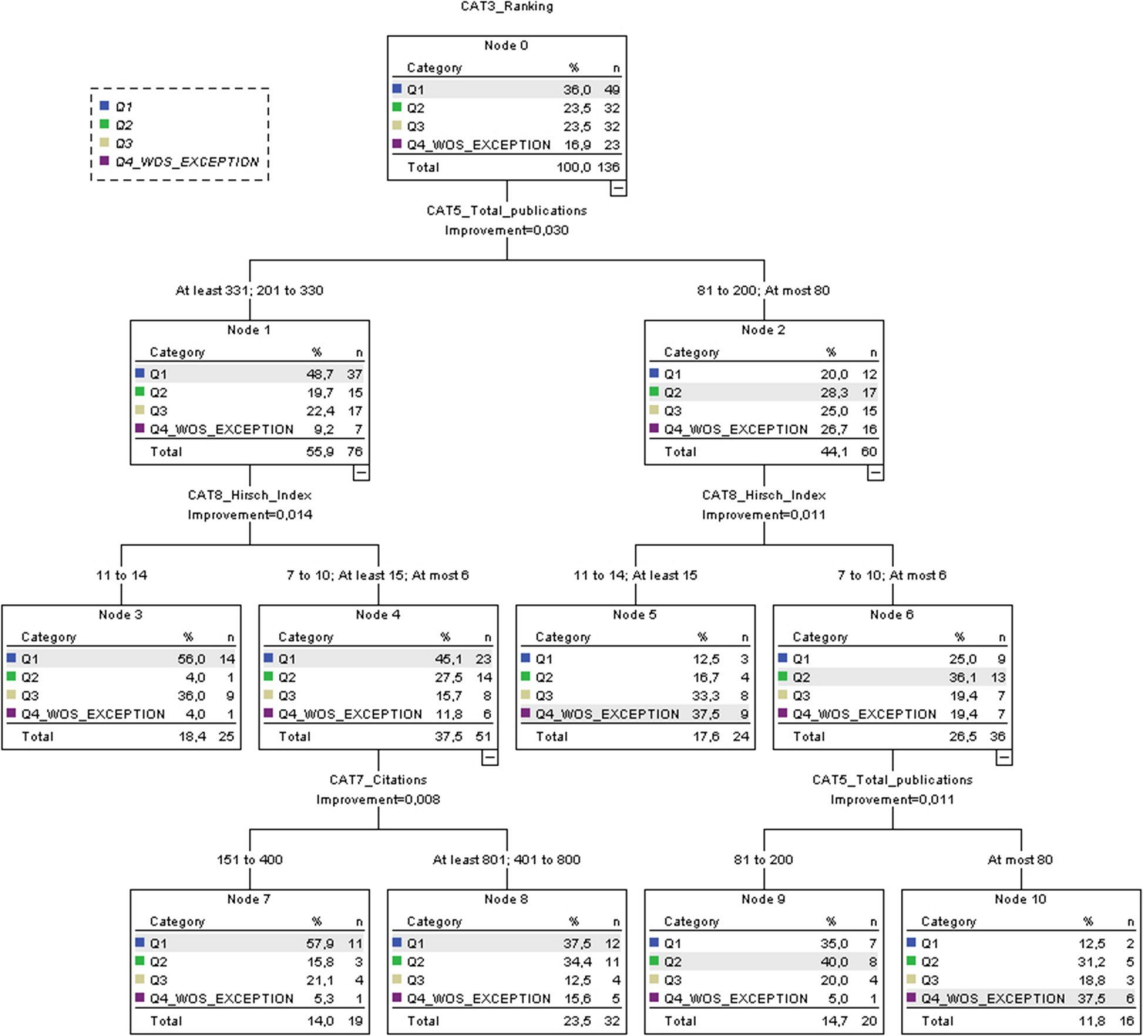
The developed CART model for the scientometric features

It was attempted to develop a multivariate CART decision tree to formulate a predictive model explaining journal ranking with the combination of scientometric variables presented by Fig. 2. The tree begins with promising results when classifying the articles by lower and higher publication performance of (co-)authors; however, further splits did not provide intuitive results. The classification accuracy of the CART is 55.5% indicating unfavorable model performance. Since both bivariate and multivariate results indicate the lack of relationships, *hypothesis 2 is rejected*.

The research has been pursued with clustering in order to test the validity of hypothesis 3. Since our database consists of categorical variables, traditional multivariate clustering techniques (such as hierarchical methods, k-means clustering) cannot be applied to cluster the publications, as their distance definition presumes continuous variables which is not the case here. For that reason, the SOM has been employed to create clusters using the 11 descriptive variables. Records have been grouped so that records within a cluster tend to be similar to each other, whereas records in different clusters are dissimilar.

**Fig. 3:**
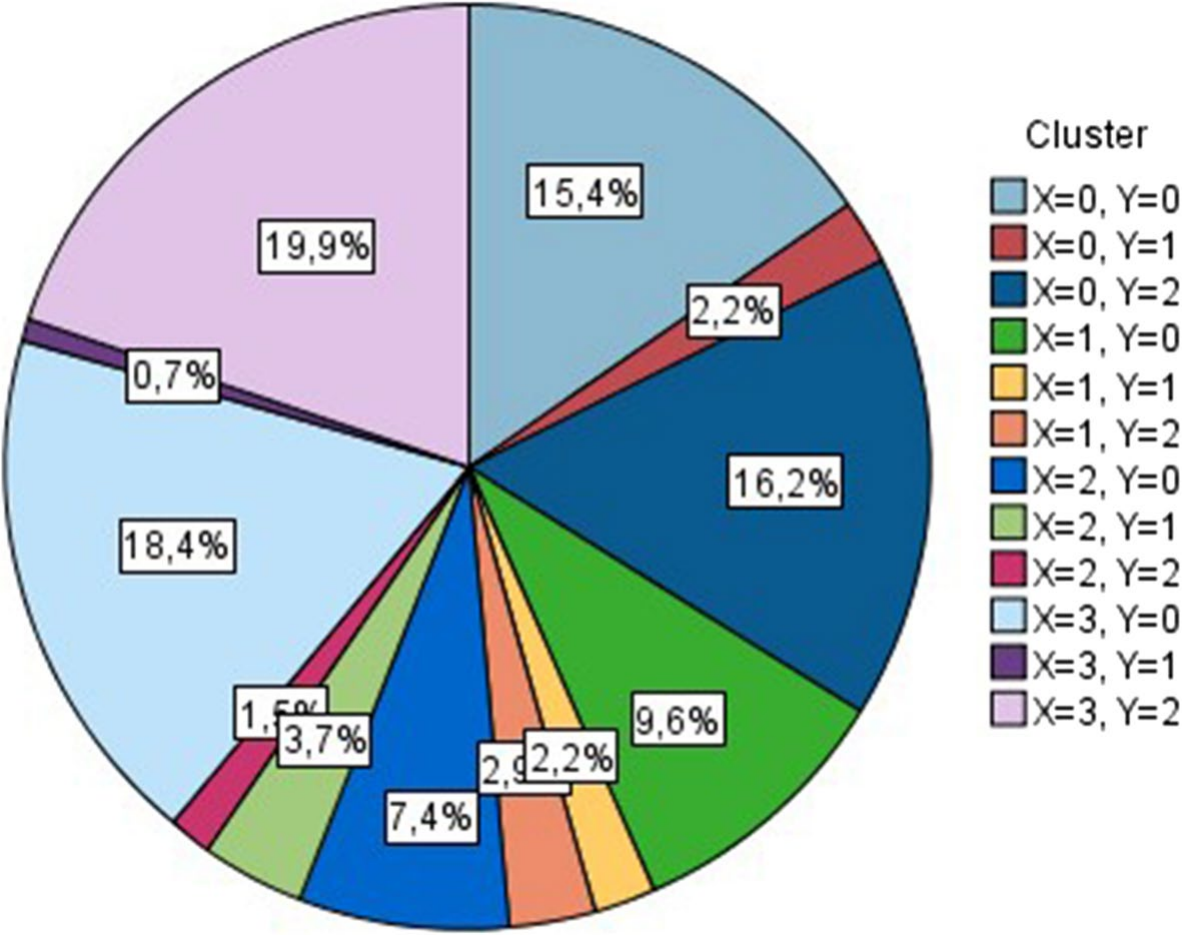
The distribution of clusters in the SOM model

The SOM algorithm has managed to identify 12 clusters within the database that can be plotted onto a two-dimensional (4×3) map. Figure 3 presents the distribution of clusters colored by the *X* and *Y* coordinates on the map.

**Fig. 4:**
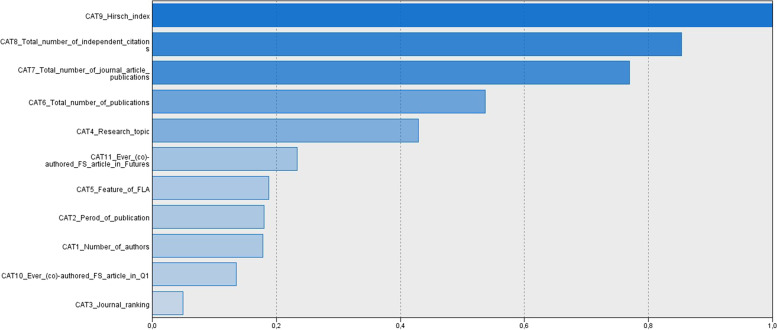
Importance of clustering variables in the SOM model

Although SOM is not a parametric method where statistical testing would show the significance of variables in the formed clustering, it is still possible to demonstrate the relative importance of each variable via sensitivity analysis. Figure 4 analyzes the importance of variables when formulating clusters. Results demonstrate that the Hirsch index and independent citations of the best Hungarian futurist (co-)author are the two strongest clustering variables.

**Fig. 5:**
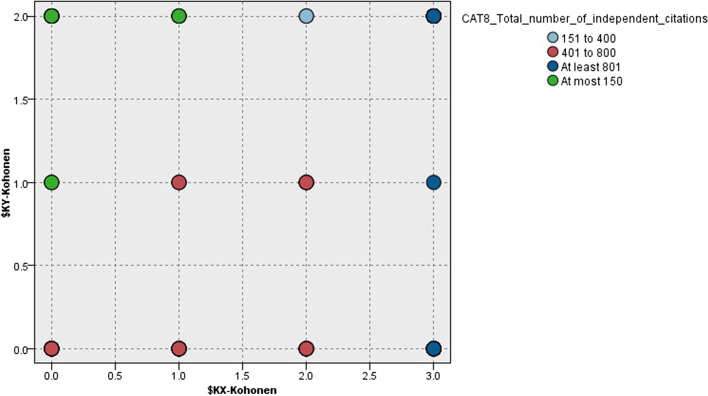
Citation-based clusters according to the SOM model marked by the number of independent citations

Making use of the SOM model, it is possible to plot the clusters based on the color-scaled legend. Figure 5 shows that articles (co)-authored by the most (>801) cited Hungarian futurists are located on the right column marked dark blue. Articles (co)-authored by the second best (401–800) cited Hungarian futurists can be found in the low-middle zone of the map marked red. Articles (co)-authored by the least cited Hungarian futurists are plotted on the top-left zone marked green. Results can be regarded as spectacular.

Hence, *hypothesis 3 is accepted*, as results demonstrate that visual clustering of journal articles where Hungarian futurists were able to publish can be accomplished by considering the independent citation profile of the “best” Hungarian (co-)author of a given article.

### The story of FS in Hungary in light of international journal articles

Futures research has been conducted in Hungary since 1968: the same year when the Club of Rome called for research into the future of the world. Kovács and Nováky [81] provided an extensive review about the organizational development, as well as the scientific results achieved in the first 25 years, whereas Nováky [105] summarized the educational and methodological experiences of that period. Assessing the advances afterwards, Nováky et al. [116] argued that there were two stages of development: the first was the vertical deepening and horizontal expansion of the field, whereas the second could be described as the theoretical and methodological renewal as well as broadening focus on practical issues. Beyond international impacts and linkages, local contextual factors, and specific opportunities also contributed to place Hungarian FS onto the global map of the discipline [79].

### Results of Hungarian FS in publications having theoretical focus

In the field of *theoretical research*, Hideg [50] argued that foresight ability represented the main difference between ordinary and emergent complex systems and presented methodological approaches to explore the possible future behavior of complex social systems. From the ideas of dynamics, cultural-interpretative FS emerged making use of negative feedbacks [47]. Hideg [49] analyzed *evolutionary* and *critical FS* as rival paradigms and concluded that both contributed to the development of futures methods. Kiss [69] conceptualized *postmodernism* as an epistemoligical shift having a considerable impact on FS. Hideg [52] discussed *integral futures* by the way of reconstructing futures paradigm history as responses to changing social needs for sustainability, and through the outcomes of dynamic and comparative analysis of futures paradigms, together with building cooperation between theory and practice. Nováky [106] suggested directions where FS should develop in the upcoming years. Hideg [51] analyzed the gap between theory and practice in foresight and formulated recommendations to reduce it. Kristóf [86] researched whether and how it was possible to make scientific forecasts through the investigation of the scientific-philosophical problems and methodological aspects of FS. Gáspár et al. [28] argued that the future of economics needed discussion and claimed for the materialization of social visions in the economic sphere. Nováky [109] explored the consequences of changes in the field of FS and accomplished empirical research of subjective relationships to changes.

### Results of Hungarian FS in publications having methodological focus

Regarding *methodology*, Szalai [140] carried out a multinational *time-budget research* to propose changes in attitudes combined with educational and economic improvements to promote gender equality. Nováky and Lóránt [117] provided a new methodological approach to the *cross-impact* method and adapted it to the socialist planning system, specifically to economic growth in Hungary. Alács [1] researched how uncertainties influenced the behavior of variables in a complex forecast model and how to estimate an approximate for the time horizon of forecasts.

Cagnin et al. [9] examined various methodological aspects of *future-oriented analysis*, as well as some advances to assist practitioners and stakeholders in comprehending transformations. Hideg et al. [57] developed a concept of an *interactive foresight* process and evaluated a foresight exercise concerning the development of knowledge economy through small and medium-sized enterprises. Nováky [110] depicted possible futures for Hungary for the year 2025, based on the expertise of futurists and social scientists, including the opinion of younger generations.

Nováky [104] demonstrated that small changes could have considerable consequences and might direct systems to different states that are difficult to trace, pointing to the need to explore and apply *chaos theory* in FS. Nováky et al. [112, 113] examined the time series of selected Hungarian socio-economic indicators whether they behaved chaotically, laying the foundations of empirical research of chaos. These results proved that under chaotic circumstances the prediction of the most probable future became impossible, so focus was put on devising of multiple, structurally different futures. Nováky and Orosz [119] accomplished similar empirical research two decades later and found that most of the indicators became less chaotic.

Recognizing that *action-oriented* and *participatory FS* are in close connection, Nováky [107] showed through four Hungarian case studies that properly selected groups were able to authentically evaluate issues in their environments. These served as a starting point to formulate future alternatives. Király and Miskolczi [67] discussed three subsystems of *participatory system dynamics*: group model building, participatory system dynamics modeling, and community-based system dynamics. Király et al. [66] applied *participatory systems mapping* to analyze the issue of sustainable consumption and found that its participatory potential might be limited, as it can be challenging for groups with a weaker knowledge base.

Bartha and Tóthné Szita [5] analyzed the socio-economic development of Slovakia and Hungary using the *State of the Future Index* (SOFI) and identified the areas where both countries should develop. Using the two-axes method, Retek [125] developed an *online scenario building* platform enriched by a text mining package, which facilitated that the entire scenario building process could be accomplished. Hideg et al*.* [55] carried out *horizon scanning* applying Osgood’s semantic differential scale to map the possible changes in natural and social systems until 2050 and to show possible interactions between the changes to enable formulation of research tasks useful for practice.

Since Finnish, Estonian, and Hungarian belong to the Finno-Ugric language family, the three countries traditionally maintain good relationships. It is well known that Finland has achieved outstanding results in the field of FS. Nováky and Monda [118] evaluated the respective organizations and the foresight system of Finland and formulated proposals to develop a strategy to utilize the Finnish experiences in Hungary. Márton [92] analyzed the education and practice of FS in Estonia and found that it achieved its first successes after the political change of the early 1990s.

### Results of Hungarian FS in publications dealing with science, technology, and innovation policy

Hungary launched its first *technology foresight program* in 1997, which was a holistic foresight exercise based on panel activities and a large-scale Delphi survey, with a strong emphasis on socio-economic needs [37]. A wide range of policy conclusions and methodological lessons were drawn. Accession to the European Union (EU) in 2004 gave a new impetus to conduct foresight projects in Hungary. By that time, several approaches had been compared to highlight good practices and methods that were likely to be useful in different environments [21].

Havas [36] argued that a sound, coherent innovation policy was one of the cornerstones of an overall development strategy, if a country was to excel, which was missing in the 1990s, and the lack of an explicit innovation policy hindered long-term development. Havas [40] analyzed research, development, and innovation collaborations between businesses and academia and found that due to different needs of firms more refined policy measures should be devised to promote collaboration more effectively.

*Responsibility for the future* is closely linked to innovation and responsibility of entrepreneurship [108]. The responsibility of futurists lies in elaborating, communicating, and implementing future alternatives together with convincing people that essential changes have to be accepted by the public. Experiences of four strategic foresight projects conducted in Hungary enabled to enhance strategic foresight by defining the role and responsibility of professional futurists and lay people [120].

Hronszky [61] outlined the features of *technology assessment* and introduced the claim for technological citizenship in formulating technological development policies. Havas et al. [42] put foresight into a broader context of policy making, with a particular emphasis on impacts on *innovation policy*, and reviewed the evaluation results of several national foresight programs. Since FLA can influence innovation systems in various ways to a significant extent, a better understanding of the “fit” between forward-looking activities and the innovation policy governance sub-system is needed to design more effective foresight or other FLA projects [44].

Havas [41] assessed the widely used methods for measuring business innovation, as well as their relevance to measure social innovations and offered multiple methodological and policy conclusions. Hronszky and Kovács [63] recognized that innovation was a networked phenomenon where the main issues happened in a globally connected world through various varied intersections. Daimer et al. [13] examined how societal aspects are considered in research and innovation activities in four scenarios fundamentally different from current practices.

### Results of Hungarian FS in publications having political and economic transition focus

Since Hungary faced a *transition* from a one-party state to a parliamentary democracy and from a centrally planned economy to a market economy, it has become a lengthy and intense discourse when and how the transition might end [134]. According to Szentes [142], beyond the system transformation, relinking to the world economy and adjustment to its rules norms became essential in the era of accelerating globalization. Mészáros [102] revealed how the need for change emerged in the economic management system.

Much earlier Hoós [58] argued that the Hungarian economy should be set on a new growth path quite different from the earlier one. Kovács [78] envisaged a lasting transition for further development. Szentes [141] warned against copying the models of advanced countries by presenting several counterarguments. Hoós [59] argued that a government in the market economy should not deal with the perfectly known past or present situation, but with the future. Havas [35] assessed the impacts of the ongoing transition on the diffusion of laser technology in Hungary. Gáspár and Nováky [30] recommended a transition paradox for methodological renewal in FS.

After completing the first round of transition in the 1990s, Hungary had to consider what role to play in the globalizing learning economy. In the former centrally planned economies there was a general vision to catch up to the developed West [129]. The evolving new world order required integration theory and new regionalism [25].

### Results of Hungarian FS in publications dealing with globalization and international economics

*Tóth* [146] recognized that technology-based *globalization* would take place anyway and fighting against technical globalization was like tilting at windmills. Since the economic processes of globalization and technological developments changed the business environment, and cooperation among firms can be an answer to the changing economic environment and to the intensifying market competition, S. Gubik [126] highlighted new opportunities for small and medium-sized enterprises in Hungary. Even if the relationship between globalization and the environment emerged mainly due to increased economic integration, globalization also meant a conceptual change in the way one thinks about the environment [147].

The turn of the century was in the center of interest [145]. Simai [135] warned not to forget the critical trends identified in the twentieth century, since new challenges for the twenty-first century would emerge from the complex historical heritage. Simai [133] argued that the corroboration of global governance was needed, since countries and other actors having common problems and mutual interests might better shape and influence global developments in an orchestrated manner.

Vág [149] evaluated the most important computerized *world models* and concluded that methodological concepts and trends promised new theoretical synthesis of world modeling. Hronszky [62] criticized the instrumentalist approaches to technological growth. Simai [131] anticipated growing inequalities for the following 50 years. Even if states would not lose their significance, economic realities and necessities create a priori determined circumstances to which a state is bound to adjust itself [132]. Simai [136] discussed the role of organized religion and communities of believers in shaping world orders and influencing their function. Kristóf [87] forecasted the risk of sovereign defaults considering the increased economic vulnerabilities due to the COVID-19 crisis.

While a constructive solution was needed to global political and social problems, it was not in the hands of politicians, as the crisis created an illusion that new social and political problems could be solved in the framework of the old, pre-global structures [71]. Hence, it became important to understand the root causes of the problems in the coexistence of globalization and modern nation state [70].

### Results of Hungarian FS in publications dealing with sustainability

Nature and society have always been in a secret dialogue with each other, forming visible and invisible oppositions, taking over the role of the other and embodying universal values for the other side [72]. When highlighting the needs of the future and present, *sustainable development* concepts often neglect how these needs are born: that is, by the relationship between future and present generations [29]. *Balázs and Gáspár* [2] revealed that the current generation lived on the cost of the future’s generation, “consumed” their futures, determined their actions and did not give them the freedom of choice and forced them to fulfil their obligations imposed on them. Király et al*.* [65] concluded that the failure to implement sustainable policies was not merely due to the fact that successful political leaders lacked systems intelligence or foresight, but that their political survival ambitions influenced their judgements.

Gáspár and Ramos [31] argued that the development of WFSF in the longer term would require a generational approach incorporating more pronounced youth and student participation. Tóthné Szita and Buday-Malik [148] evaluated the progress of a Hungarian county and identified changes to move towards sustainable development. Zajáros et al. [153] accomplished a life cycle *sustainability assessment* project for wastewater management. Köves et al. [85] discussed the role and responsibility of business organizations in a sustainability transition in a thought-provoking hypothetical construct: the cuvée organization.

Dombi et al. [17] carried out a *socio-economic metabolism* examination to compare household-level material flows and stocks to economy-wide material inputs and explore factors affecting the level of metabolism [34] at the household level. According to Dombi et al. [19], there is no incentive to reduce the material stock accumulation in the future under the current economic conditions; however, urbanization and the increasing share of the service sector in economic output might support the sustainability transition due to its lower material input and emissions, while accelerating economic growth at the same time [15]. The management of material stock is central for reducing emissions and for optimizing natural resource use [16]. Dombi [14] developed a forecast model to estimate the capital stocks of agriculture, industry, and services both in physical and in monetary dimensions. Dombi et al*.* [18] argued that rationalization of consumption, more efficient energy usage, and a new energy structure should be achieved to shift the structure of energy system towards sustainability.

Young et al. [152] developed *design orienting scenarios* and formulated exact proposals for product-service systems using stakeholder creativity workshops to illustrate a range of environmental futures and thereby support the design, business, and policy-making processes. Kiss et al. [73] applied participatory systems mapping to identify system boundaries for sustainable consumption and uncovered causal relationships among the determining factors of sustainable consumption. Balázs et al. [3] analyzed case studies to understand the spectrum of localized, alternative food futures.

Király et al. [64] and Köves et al. [83] conducted a backcasting project on sustainable employment in Hungary and demonstrated that underlying theories on social change operated and influenced the project. Balázs et al. [4] accomplished a participatory agenda-setting exercise for green services in Hungary to initiate social dialogue around green care in the country. In a recent backcasting exercise, Köves and Király [82] attempted to reorient marketing communication to support sustainability transitions in society. Within the framework of a similar backcasting project, Köves et al. [84] anticipated the sustainable future of sports and identified potential intervention steps that can lead towards the imagined normative states. Csákvári et al. [12] assessed future environmental and biological conservation issues and set priorities for Hungary in line with global and European biodiversity strategies.

### Results of Hungarian FS in publications dealing with regional foresight

Korompai [76] claimed that the earlier attitude towards *regional development*, characterized by the orientation towards more extensive and intensive use of existing local resources, had to be modified and laid the foundations for regional strategies with futures methods. Kovács and Korompai [80] analyzed multiple development scenarios together with setting international urban development trends to elaborate the development strategy for Budapest. Recognizing that the Hungarian rural development planning showed the lack of future orientation, Korompai et al. [77] demonstrated how to provide useful information for the planning of rural development for 20–30 or even more years ahead, by applying scenarios, efficiently highlighting the unexpected *weak signals* of the driving forces.

Péti [124] examined the presence of general and territorial sustainability in regional development programs and proposed a new planning and assessment system based on a set of regionally legitimate sustainability values. Málovics [33] found that it is a challenging task for participatory action learning to enable and empower community members to make pragmatic and sustainable changes in their lives.

### Results of Hungarian FS in publications dealing with future orientation examinations

A definition and typology of *future orientation* were elaborated by Nováky et al. [114, 115] to conduct empirical research on the capacity of human foresight in different stakeholder groups. Initial results revealed dissonance in future orientation residing in the discrepancy between interest in the future and actions performed for the future [54]. Hideg and Nováky [56] completed a comparative analysis of findings in the mid-1990s and 2000s. Hideg [48] recommended that university students should be trained to be able to apply a wide variety of futures methods to help people and various organizations develop their future orientation.

Nováky and Gáspár [111] emphasized the role of youth for a less selfish future. Molnár and Vass [103] compared the future orientation of German and Hungarian youth and explored pessimistic characteristics in their attitude. Horváth and Nováky [60] elaborated a predictive model of future orientation based on current and future norms, future interests, and concerns. Nováky and Várnagy [121] went beyond discovering the future orientation of youth and elaborated complex social future alternatives.

### Results of Hungarian FS in publications dealing with the future of higher education

Regarding the *future of higher education*, Havas [38] offered a set of cascading visions for universities, by considering the overall rationale of EU policies and initiated meaningful dialogues among stakeholders. Havas [39] argued for a systematic *prospective analysis* for the future of the higher education by highlighting the potential changes of broader settings, in which universities operate, as well as their impacts on higher education together with the likely impacts of different policy options. Duma and Monda [20] analyzed the possible future impacts of information and communication technology tools on education applying *futures wheel*, giving a basis for building four scenarios.

Pataki et al. [122] conducted a *participatory backcasting* project concerning the future of higher education involving lecturers and students. Toarniczky et al. [144] accomplished *transformational learning* at a leading business school in Hungary and revealed how it might be supported at various levels.

Since *experimental foresight* is closely allied with *experiential futures*, Woodgate and Veigl [151] completed an experimental foresight on the potential futures for universities in Norway, where the authors distinguished the two in such a way that experiential referred to the way the foresight was conducted, whereas experimental to what was conducted.

Király and Géring [68] focused on the social expectations towards higher education institutions and highlighted their advantages, disadvantages, and possible future consequences. Gáspár et al. [24] showed that modern futures methods in education not only improved knowledge, skills, and attitudes, but became useful to develop the competences of the individuals to participate in collective future shaping actions and enhance their feeling of autonomy and responsibility for future.

### Results of Hungarian FS in publications dealing with corporate future

Gáspár [23] analyzed through a Hungarian case study how to construct futures in the *corporate strategy formulation* practice. Beyond the consciously applied foresight techniques, managerial activities also led to foresight-generating actions. Gáspár [27] offered a new perspective, that is, “Strategia Sapiens” for *strategic foresight* based on nurture theory, which strengthened the incorporation of cultural responsibility and the intergenerational view of strategic foresight.

Gáspár [22] studied the interpretations of the future and time from the perspective of strategy research and practice, respectively. Gáspár [26] formulated a new interpretation of strategy much closer to social foresight and looked at path dependence and path creation as two pillars of strategic activity. In addition to corporate foresight, machine learning-based modeling represented a key research focus to forecast *corporate failure* [88, 89, 150].

Cooperation among the so-called Visegrad Four Group (Poland, Czechia, Slovakia, and Hungary) has been traditionally significant for Hungary. Sacio-Szymańska et al. [128] outlined the context for *business foresight* in these four countries and developed scenarios with entrepreneurs aimed at bringing futures knowledge and techniques to managers. For companies, there are different motivations for applying foresight [75].

To gain insight what shapes the social motivations of entrepreneurs in Central and Eastern Europe and how to strengthen these motivations to make social entrepreneurship more effective, Bartha et al. [6] studied the entrepreneurial motivations of university students and uncovered the influencing factors behind the motivation factors. S. Gubik [127] examined further the shaping factors of entrepreneurial ambitions that enabled to develop policies and university practices to increase students’ entrepreneurial intention and thus entrepreneurial activity.

### Results of Hungarian FS in publications dealing with the future of health

Digital health has been reshaping healthcare systems since the beginning of the twenty-first century. It not only refers to technological transformation, but it also fundamentally changes the physician-patient relationship and treatment circumstances, as well serves as a guide to prepare for the future technologies that will have to be implemented in everyday practices and in the health management of patients [32, 94, 96, 100, 101]. Artificial intelligence and machine learning show potential for diagnosing, managing, and treating a wide variety of medical conditions [98, 139]. Meskó et al. [99] carried out qualitative, semi-structured interviews with opinion leader empowered patients to explore their experiences and beliefs about technologies, and how they see the future, and identified four major themes emerging from their experiences. *Meskó* [97] discussed scenarios about the future of digital health and aimed at addressing the major challenges around that, even thinking of the first human mission to Mars [95].

## Conclusions

This comprehensive literature review shows a colorful research landscape, with Hungarian researchers dealing with various topics and achieving scientific results in advancing theoretical and methodological development of FS, as well as in applying futures methods.

In the field of theoretical development, Hungarian futurists defined the relationship between FS and other disciplines from the viewpoint of philosophy of science, explored and analyzed the positivist, evolutionary, and critical directions of FS, defined and evaluated future paradigms, enriched the features of integral futures, interpreted and empirically measured future orientation, connected critical future trends with global challenges, such as postmodernism with FS, recognized the increasing importance of participatory FS in shaping future alternatives, and in accomplishing the positive ones in an action-oriented way, demonstrated the responsibility of futurists and emphasized the substance of methodological purity and rigor, and the acceptance of participatory processes.

Regarding methodological development, Hungarian futurists finetuned and successfully applied the cross-impact method; studied the behavior of complex, dynamic systems; recognized the relevance of chaos theory in FS; examined Hungarian macro processes from chaotic viewpoints; developed methods to consider complexity, participation, and alternativity to draw acceptable multiple futures for Hungary by involving the examination of stability and forces shaping the future; adapted and measured reliability in forecasts; applied participatory backcasting, digital tools, and big data methods in shaping the future of health and education; and accomplished scenario building in an interactive environment through online communication. Their focus on sustainability together with its research methods is becoming ever stronger and more visible.

Hungarian futurists also applied methods elsewhere developed in Hungarian circumstances: elaborated SOFI for Hungary, adapted the multiple factor approach of technology foresight, defined the social dimensions of entrepreneurial motivations, interpreted socio-economic metabolism and identified their drivers on the household level, employed horizon scanning to anticipate the environmental situation of Hungary in 2050, and forecasted corporate failure.

Our review reveals that Hungarian futurists repeated certain empirical research efforts (such as future orientation or chaotic behavior examinations) from time to time to evaluate changes. It also underlines the importance of international collaboration. Hungarian futurists have followed international good practices, monitored and assessed their own level of development, and contributed to advancing the discipline internationally.

Within the framework of the systematic literature review, a statistical-based hypothesis examination has been performed to test what descriptive variables of articles and (co-)authors are in relationship with the quality of articles expressed by journal ranking. It is concluded that both the period of publication and the research topic are significant in this aspect; however, neither scientometric feature of (co-)authors shows significant association with journal ranking. Results have demonstrated that Hungarian futurists with lower publication and citation track records can publish in high-ranking journals, and for the time being sustainability, future of health, and methodology-driven papers have the best chance to be published in Q1 journals. Visual clustering of the examined publications has been completed by SOM, where results have demonstrated that the Hirsch index and independent citations of the best Hungarian futurist (co-)author of the articles are the two strongest clustering variables.

With this review, we also acknowledge the crucial impacts of international partnership and cooperation among futurists and would be pleased to read similar reviews on the evolution of this research domain in other countries. It is hoped that the systematic review of Hungarian FS results might launch a process leading to a comprehensive picture on the global results of FS and draw the attention to what problems should be solved by FS for humanity.

## Data Availability

Not applicable.

## Abbreviations

APF: Association of Professional Futurists
CART: Classification and Regression Trees
EU: European Union
FLA: Forward-Looking Activities
FS: Futures studies
HAS: Hungarian Academy of Sciences
MTMT: Hungarian Scientific Bibliography (Magyar Tudományos Művek Tára in Hungarian)
SOFI: State of the Future Index
SOM: Self-Organizing Maps
UNIDO: United Nations Industrial Development Organization
WFSF: World Futures Studies Federation
WOS: Web Of Science

## Footnotes

1 If more than one Hungarian futurist (co-)authored an article, the author having the best scientometric parameters was selected for further analysis.
